# Additional effects using progestins in mares on levels of thyroid hormones and steroids in neonates

**DOI:** 10.1590/1984-3143-AR2023-0029

**Published:** 2023-12-18

**Authors:** Ana Carolina Rusca Correa Porto, Mariana Abreu Redoan, Cristina Oliveira Massoco, Priscila Viau Furtado, Claudio Alvarenga Oliveira

**Affiliations:** 1 Universidade de Sorocaba, Faculdade de Medicina Veterinária, Sorocaba, SP, Brasil; 2 Fazenda Santa Rita, Piracaia, SP, Brasil; 3 Departamento de Patologia, Faculdade de Medicina Veterinária e Zootecnia, Universidade de São Paulo, São Paulo, SP, Brasil; 4 Departamento de Reprodução Animal, Faculdade de Medicina Veterinária e Zootecnia, Universidade de São Paulo, São Paulo, SP, Brasil

**Keywords:** altrenogest, progesterone, cortisone, equine, pregnancy

## Abstract

The risk of pregnancy loss in mares leads to the use of exogenous hormones to help pregnancy maintenance. The objective was to evaluate the proportion of thyroid hormones and steroids in neonates, in the following postpartum period, born to mares fed with synthetic progesterone and to verify the existence of a correlation between the level of progesterone between mother and neonate. Twenty-seven mares and their foals were used. The animals were divided into 5 experimental groups: group 1 (control, without hormonal supplementation), group 2 (random samples fed to 120 days of pregnancy with long-term progesterone), group 3 (mares fed with short-term progesterone as of 280.º day of pregnancy), group 4 (mares fed with long-term progesterone as of 280.º day of pregnancy) and group 5 (mares fed with synthetic hormone [altrenogest] as of 280.º day of pregnancy). The animal’s blood collection took place immediately after parturition for the hormonal measurement. The hormones measured in neonates were total T3, free T4, TSH, progesterone and cortisone. In mares, only levels of progesterone. The groups of neonates showed no difference on levels of total T3, free T4, TSH and progesterone. There was no difference on levels of progesterone in mares among the groups. Neonates from groups 4 and 5 had higher and lower cortisone levels, respectively. No neonate showed clinical change. There was also no correlation between levels of progesterone in mares and foals. Thus, hormonal supplementation with long-term progesterone as of 280 days of pregnancy leds to an increase in the neonate's cortisone levels, in the meantime, supplementation with altrenogest as of 280 days of pregnancy caused a decrease on cortisone levels in foals, despite clinical signs have not been observed on these animals.

## Introduction

The high economic cost of a pregnancy by embryo transfer in mares and the great value of the live foal make professionals fear the failure of pregnancies and adhere to the use of exogenous/synthetic progesterone (Allen, 2001; DeLuca et al., 2011). There is no proven method to select which mares should be supplemented, causing the medication to be applied indiscriminately. Massive supplementation of the herd has not shown changes in rates of early pregnancy loss and percentage of alive foals for the last 12 years; besides, the period of greatest loss (15-42 days of pregnancy) remains similar in groups with and without exogenous progesterone (Rose et al., 2018).

In women, it has been described that exogenous progesterone supplementation can suppress the transcription of some genes, including some which are responsible for the production of cytokines and the proliferation of T cells after the introduction of antigens, which would impact the susceptibility to pathogens (Bamberger et al., 2000; Koubovec et al., 2004; Huijbregts et al., 2013). Although many studies have demonstrated the anti-inflammatory effect of progesterone in the context of reproduction, there is little understanding about its cellular and molecular actions that can generate immunomodulation (Goddard et al., 2013)

In fetus, in a period close to the birth, the adrenal gland becomes able to produce cortisone (Silver, 1990; Fowden and Silver, 1995; Fowden et al., 2012). Since adrenocortical activity increases prepartum, it is expected to find high levels of plasmatic cortisone in newborns in their first hours of life (Fowden et al., 2012; Silver et al., 1984; Lester, 2005). Foals that have undergone stress from disease, such as sepsis, have higher levels of total cortisone, and this value can be higher on those that did not survive (Panzani et al., 2009; Hart and Barton, 2011).

Another process that occurs close to the birth, is an increase in thyroid hormones, namely triiodothyronine (T3), thyroxine (T4) and even thyrotrophin (TSH) (Jeffcott et al., 1982; LeBlanc et al., 2004). Thyroid hormones have a direct action on fetal metabolism, especially on oxygen consumption, glucose and thermoregulation (Forhead and Fowden, 2014). Horses have one of the highest levels of flowing thyroid hormones, with the level at birth being 10-20 higher than adults (Knottenbelt et al., 2004). Along with cortisone, triiodothyronine (T3) seems to be critical for lung maturation (Forhead and Fowden, 2014; Barker et al., 1991; Ramminger et al., 2002). Thus, the effects of hypothyroidism in neonates are much more severe than in adults since the actions of thyroid hormones are crucial for the development of nervous and skeletal systems (Frank et al., 2002).

Knowledge about the consequences of the use of synthetic hormones in foals of mares with supplementation is almost non-existent, despite the evidence that altrenogest (derived from 19-norethisterone steroid), the most currently used, may be able to cross the placental boundaries, and its high levels described as causing problems in the newborn's life (Neuhauser et al., 2008, 2009; Palm et al., 2010). Thus, the objective of this study was to evaluate the hormonal levels of newborns in the immediate postpartum of mares submitted to different supplementation protocols with synthetic progesterone and to verify the correlation between the mother's progesterone levels with the newborn's hormonal levels of progesterone.

## Methods

This project was approved and developed in accordance with the guidelines of the Ethics Committee on the Use of Animals of the Faculty of Veterinary Medicine and Zootechnics of the University of São Paulo, under protocol number 9874130317/2019.

Twenty-seven pregnant mares and their foals were selected through a suitable sample, all of them being of the Mangalarga Marchador breed, from a owner in São Roque, São Paulo, Brazil.

The animals were kept in coast-cross paddocks where they also received balanced feed twice a day and water uninterruptedly. All health protocols were carried out as recommended for horses and their respective stages of life. Mares were vaccinated for rhinopneumonia (Vencofarma, Brasil®) in fifth, seventh and ninth pregnancy months. Still, in the ninth month were applied tetanus, encephalomyelitis and influeza vaccines (Vencofarma, Brasil®). The animals were used exclusively for the experiment. All mares were inseminated with the same stallion during 2014-2015 breeding season and submitted to the same management. The pregnancy was monitored, every 30 days, with evaluation of fetal and placenta viability, and the births attended to ensure a promptly intervention in case of problems.

Soon after birth, all necessary parameters to evaluate the physical health and viability of the foal were measured and recorded, or possible complications arising from the intervention of the experiment.

Five experimental groups were defined, namely:

- GROUP 1 (control): pregnant mares that were not submitted to supplementation with exogenous hormones (n=4);
- GROUP 2 (treatment 1): Supplemented non-cycling receivers (induced to an artificial cycle). They received estradiol cypionate for three days after the embryo donor's ovulation. On the fourth day, 1500 mg of long-lasting natural progesterone (P4-LA) was administered. Once the embryo was retrieved on the eighth day after the donor's ovulation, another application of P4-LA in the receiver and the embryo transfer was performed. When pregnancy was confirmed, P4-LA was applied every 7 days until 120 days of pregnancy (n=4);
- GROUP 3 (treatment 2): Hormonal supplementation (P4-CA). As of 280 days of pregnancy, the mares received 225 mg of short-term natural progesterone once a day until the foal's birth (n=5);
- GROUP 4 (treatment 3): Hormonal supplementation (P4 - LA). As of 280 days of pregnancy, the mares received 1500 mg of long-term natural progesterone (P4-LA) once a week until the birth of the foal (n=5);
- GROUP 5 (treatment 4): Hormonal supplementation with hormone derived from 19-norethisterone steroid (E-19N) (altrenogest). As of 280 days of pregnancy, the mares received 300mg of (E-19N) once a week until the foal's birth (n=4).

To perform serum hormone dosages in mares and foals, plasma was used and collection was carried out immediately after parturition, in 10 minutes maximum.

The dosage of progesterone in mares was performed using the radioimmunoassay (RIA) technique, following the protocol described by the manufacturer (Beckman Coulter, Immunotech/USA).

The levels of triiodothyronine (T3) and thyroxine (T4), progesterone and cortisone in foals were also determined using the Radioimmunoassay (RIA) technique and different commercial diagnostic kits were used (Coat-A-Count - Diagnostic Products Corporation/USA kit was used to T3 and T4; Beckman Coulter, Immunotech/USA kit was used to progesterone and cortisone). For the thyrotrophin test (TSH), the enzyme-linked immunosorbent assay (ELISA) was performed.

The data obtained were analyzed using Minitab®19 (Minitab, 2019).

The Shapiro-Wilk test was used to verify the normality of data distribution. For data that did not show normal distribution (free T4 and TSH) the Kruskal-Wallis test was used to compare the average. For variables with normal distribution (mother’s P4 and newborn’s P4, total T3 and cortisone), Analysis of Variance (ANOVA) was performed to detect the average difference of each hormone among groups, with a significance level of 5%. Thus, in cases where ANOVA detected significant interaction among groups (p<0,05), the Tukey's range test was applied. The Dunnett's test was also performed, adopting a 95% confidence interval, when applicable.

Pearson's correlation was used to calculate the relationship between progesterone from the mother and progesterone from the neonate.

## Result

When comparing the plasmatic concentration average of TSH, T3, T4 and progesterone, in newborn foals from the groups of the experiment, no statistical difference was observed (p=0,46) (p=0,71), respectively. When comparing cortisone average among the groups, a difference was observed (p≤0,001), and after performing the Dunnett's test, it was found that cortisone average in groups 4 (11,10) and 5 (1, 16) was higher and lower when compared to the control group (5.88), respectively (Table 1).

**Table 1:** Means and standard error of hormonal dosages of TSH, T3, T4 and progesterone in newborn foals and adult mares in the five experimental groups.

**Foals**	**Group 1**	**Group 2**	**Group 3**	**Group 4**	**Group 5**	**≠1x2**	**≠1x3**	**≠1x4**	**≠1x5**
	**Average (se)**	**Average (se)**	**Average (se)**	**Average (se)**	**Average (se)**				
Thyrotrophin (TSH) (ng/mL)	1.34 (0.78)	3.15 (1.61)	1.30 (0.71)	2.18 (1.12)	0.32 (0.07)	135%	-3%	63%	-76%
Triiodothyronine (T3) (ng/mL)	4.32 (0.58)	3.49 (1.02)	5.27 (0.80)	4.81 (0.56)	4.96 (0.38)	-19%	22%	11%	15%
Thyroxine (T4) (ng/mL)	6.51 (0.72)	6.67 (1.60)	5.51 (0.37)	12.71 (2.58)	6.28 (0.50)	2%	-15%	95%	-4%
Progesterone (ng/mL)	16.04 (4.06)	17.65 (6.20)	10.10 (2.46)	12.98 (3.42)	6.05 (1.50)	10%	-37%	-19%	-62%
Cortisone (ng/mL)	5.88 (0.77)	11.15 (1.08)	7.89 (2.49)	11.10 (0.75)	1.16 (0.26)	90%	34%	89%	-80%
**Mares**									
Progesterone (ng/mL)	15.88 (3.25)	11.25 (8.96)	4.80 (1.59)	4.40 (0.67)	26.00 (15.79)	-29%	-70%	-72%	64%

se = standard error; ≠differences calculated by (treatment group - control group)/control group*100.

In the analysis of the comparison of the dosage of progesterone in mares, it was observed that there is a significant difference among the groups (p≤0,001), however, after performing the Tukey's range test, it was not possible to identify among which groups there was this difference in the average.

Pearson's correlation, to verify if the level of natural progesterone in mares was correlated with the concentration in foals, showed that there was no relationship considering the two types of average (correlation coefficient: 0,208).

## Discussion

Our study evaluated the level (in the immediate postpartum period) of some important hormones for the development of the neonate, since few studies have evaluated the effect of supplementation with synthetic progesterone in horses (Neuhauser et al., 2008, 2009; Palm et al., 2010; Jackson et al., 1986; Shoemaker et al., 1989; Ousey et al., 2002; Fedorka et al., 2019; Müller et al., 2019).

Changes in thyroid hormones levels in the intrauterine period can trigger permanent changes in the structure and function of tissues and organs (Forhead and Fowden, 2014). The investigation of thyroid dysfunction in horses has many difficulties due to multisystemic and non-specific effects (Messer et al., 1998). A study compared the levels of thyroid hormones in mares and neonates of different properties in relation to the reference values ​​and found a high percentage of animals with T3, T4 and TSH values ​​below the normal range, although none of them presented typical signs of apparent gland dysfunction (Messer et al., 1998). In our study, in addition to not having suggestive physical characteristics of thyroid disorders, neonates from different groups did not show any difference considering the levels of total T3, free T4 and TSH.

In relation to TSH, among the few studies that measure it in equine neonates, it has been observed that the hormone level is the same for foals and adult horses; on the other hand, there seems to be a difference considering those who were born premature and sick in comparison to healthy ones, with the first group presenting a lower level of TSH (Berg et al., 2007; Breuhaus, 2014). Since the level of thyroid hormones in foals is higher, it is likely that there is a different functioning between the hypothalamic pituitary adrenal (HPA) axis of adults and neonates (Breuhaus, 2014). We found no difference in TSH levels when comparing the groups and there are no previous references that discuss the possible influence of exogenous progesterone supplementation on the thyroid gland and this shows that our treatment groups do not interfered in measurement.

As for the level of progesterone in newborns, we also did not find a relationship between the use of hormones in the mother and changes in the levels found in foals. Literature reports that progesterone levels are high at birth but tend to decrease over the first day postpartum and become undetectable on the second day in healthy foals (Holtan et al., 1991; Chavatte et al., 1997). However, in sick foals, levels are high at birth and remain in the following days, with the highest levels found in animals with more severe impairments (Houghton et al., 1991; Rossdale et al., 1995). When there is a higher level of progesterone in foals born to supplemented mares, a slow or non-existent response to external stimulus is expected due to the possible sedative effects that progesterone derivatives have, as they cross the blood-brain barrier (BBB) and reach the central nervous system (Madigan et al., 2012). Thus, given the fact that we measured the progesterone level in a single moment and all animals were born healthy, the decay of the plasma level was expected. Another possibility would be the inability of the dosage test to detect exogenous progesterone. This hypothesis is in the literature, considering that the type of hormonal dosage test may interfere with the measurement, leading to false results, as it would only detect endogenous progesterone; in other words, it has been indicated that the synthetic hormone (altrenogest) does not have a cross-reactivity with the immunoassay antibodies (Wynn et al., 2018). Thus, we concluded that the use of synthetic hormone in mares did not interfere with the measured levels in neonates in our experiment, or the dosage test was not able to detect exogenous progesterone, indicating a limitation of this study.

As for the level of progesterone in the mares in the postpartum period, although we found a difference among the groups in the Analysis of variance (ANOVA), in the subsequent tests (Tukey and Dunnett), this difference was not found. We believe that the large variation in data, added to the low sample size, may have contributed to this inability to detect differences in means. Corroborating our finding, other studies found no difference in the level of endogenous progesterone after using altrenogest, depending on the dosage test used (Jackson et al., 1986; Willmann et al., 2011). Another study, however, managed to differentiate the level of endogenous and exogenous progesterone after application of altrenogest, but used other techniques for measurements, such as radioimmunoassay (RIA) and Gas chromatography (GC), respectively (Ousey et al., 2002). As well as the situation of foals, we believe that for this synthetic hormone, the type of hormonal test used, radioimmunoassay (RIA), may have been inefficient in differentiation. Knowing the altrenogest immunomodulatory effects in nonpregnant mares, and understanding the limitation of hormonal dosage tests, we consider that this topic requires more sample robust studies, so that this hypothesis can be discussed in pregnant mares and their respective neonates.

The cortisone level average in neonates was lower in group five (treatment with altrenogest) and higher in group four (treatment with long-term progesterone) when compared to the control group (group one). Contradicting our results, other studies found higher plasmatic cortisone levels in those groups supplemented with synthetic hormone (altrenogest), but this difference disappeared after 15 minutes of birth. These changes in cortisone level appeared to be associated with time of delivery, insofar as those animals treated with synthetic hormone had a longer time of delivery than the control group (Neuhauser et al., 2008, 2009; Palm et al., 2010). In our study, we did not have any prolonged action delivery that could justify the increase in cortisone in group four. Another distinction between our study and the literature is the method of administration; while we use the injectable form of the product, which has a lower absorption rate but a longer blood circulation time, other works show us a daily oral administration, therefore altering the plasmatic cortisone levels at the time of measurement (Machnik et al., 2007; McConaghy et al., 2016).

A limitation of our research was not evaluating the immune response despite the existing relationship between cortisone and the immune system. Existing studies try to understand the regulation of the immune system by glucocorticoids, such as cortisone, mainly because these hormones act in multiple ways (Cain and Cidlowski, 2017). However, the inhibition of cytokine production has been considered as the most relevant phenomenon of the immunosuppressive effects of glucocorticoids (Riccardi et al., 2002). Therefore, in our study, given the variation in cortisone levels which was found in the experimental groups, somehow, we believe that it may have modulated the newborns' immune system. Such parameters would need to be measured, and the foals would need to be monitored for a longer time than we proposed in this study.

## Conclusion

There were no differences in the levels of total T3, free T4, TSH and progesterone in the neonates and in the levels of progesterone in the mares of the different groups, the small sample of animals evaluated may be the reason why no differences were observed. However, we found a higher level of cortisone in neonates in the group supplemented with long-lasting progesterone and a lower level of cortisone in the group supplemented with altrenogest. We believe that supplementation with altrenogest and natural progesterone is related to oscillations in cortisone found in neonates in the supplemented groups (groups 4 and 5). However, more studies need to be carried out to elucidate this influence.
